# β-elemene sensitizes hepatocellular carcinoma cells to oxaliplatin by preventing oxaliplatin-induced degradation of copper transporter 1

**DOI:** 10.1038/srep21010

**Published:** 2016-02-12

**Authors:** Xiaoqiang Li, Zhenhai Lin, Bo Zhang, Lei Guo, Shuang Liu, Hui Li, Jubo Zhang, Qinghai Ye

**Affiliations:** 1 Liver Cancer Institute and Zhongshan Hospital, Fudan University, Shanghai 200032, P.R.China; 2 Key Laboratory of Carcinogenesis and Cancer Invasion, Ministry of Education, Shanghai 200032, P.R.China; 3 Department of Hepatic Surgery, Shanghai Cancer Center, Fudan University, Shanghai 200032, China.

## Abstract

β-elemene, a *Curcuma wenyujin* plant extract, has been used widely as a tumor adjuvant therapeutic agent. However, how to obtain optimum therapeutic effects by combining this compound with other agents remain unclear. In this study, we found that β-elemene, which alone had little effect on hepatocellular carcinoma (HCC) cell proliferation, exerted a synergistic anti-proliferative effect in HCC cells when dosed in combination with oxaliplatin, which increased the amounts of platinum accumulation and platinum-DNA adduct significantly and augmented the oxaliplatin-induced apoptosis. Western blot and laser scanning confocal microscopy studies indicated that β-elemene enhanced the sensitivity of HCC cells to oxaliplatin by upregulating copper transporter 1 (CTR1), a major controller of intracellular platinum accumulation. In an orthotopic transplantation HCC model in nude mice, HCC tumor growth was inhibited significantly by oxaliplatin combined with β-elemene, as compared with oxaliplatin alone. Notably, CTR1 protein expression in xenograft HCC was upregulated in mice who received β-elemene treatment. Taken together, our findings show that β-elemene can block the reduction of CTR1 resulting from oxaliplatin treatment, and therefore has a synergistic anti-HCC effect with oxaliplatin by enhancing cellular uptake of oxaliplatin. The synergistic effects of β-elemene and oxaliplatin deserve further evaluation in clinical settings.

Liver cancer is one of the most highly fatal forms of cancer around the world and is more common in less developed countries. Worldwide, an estimated 782,500 new cases of liver cancer and 745,500 deaths occurred during 2012, half of them in China^1^. According to the latest statistics, the five-year survival rate for liver cancer is usually very low (10–20%) in the majority of countries, both in the East and the West^2^. Approximately 70% of patients diagnosed with liver cancer are not suitable for curative surgery due to a history of cirrhosis, a huge primary lesion, multifocal lesions, major blood vessel invasion, and/or extra-hepatic spread^3^. Hence, it is urgent to identify more effective chemotherapy agents.

Although many cytotoxic chemotherapeutic agents have been reported in the last 30 years, so far all lack convincing benefits in phase II studies^4^,^5^. A randomized, multicenter, open-label study of oxaliplatin [(trans-R,R)1,2-diaminocyclohexaneoxalatoplatinum (II), C_8_H_14_N_2_O_4_Pt] (Fig. 1a) plus fluorouracil/leucovorin (FOLFOX4) as palliative chemotherapy in HCC patients from Asia found that this regimen showed a trend toward improved overall survival, along with increased progression-free survival, although the primary end point was not met^6^. A retrospective multicenter study confirmed that gemcitabine and oxaliplatin (GEMOX) is effective with an acceptable profile of safety for advanced HCC patients^3^. Oxaliplatin as one member of mixed solution containing epirubicin, oxaliplatin, mitomycin and lipiodol participate in transcatheter arterial chemoembolization (TACE) for hepatocellular carcinoma (HCC) in China^7^,^8^. From the economic perspect, oxaliplatin, with lower price compared to sorafenib, gives more patients easily get access to chemotherapy and consequent benefit from it. Thus, a promising approach may be to investigate how to further improve the effects with combinations of existing drugs. Agents that influence the distribution, intracellular concentration, and excretion of oxaliplatin^9^,^10^,^11^,^12^,^13^ may have the potential to further improve the anti-tumor effects of oxaliplatin with less cytotoxicity.

The anti-tumor effect of platinum-based drugs is directly related to the amount of the drug that enters the cell, and nearly all platinum-resistant cells have shown a reduction of platinum entering the cell. Copper transporter 1 (CTR1), the major copper influx transporter, has been shown to control the intracellular accumulation of platinum and therefore also the cytotoxic effects of platinum-based chemotherapy drugs, such as cisplatin and oxaliplatin^14^. Several studies suggested that CTR1 transports platinum drugs via chaperones and a series of transchelation reactions in a manner similar to copper transport^15^,^16^. Therefore, the agents acting on the platinum processing pathway should be explored as combination agents^17^.

β-elemene (1-methyl-1-vinyl-2, 4-diisopropenyl-cyclohexane, C_15_H_24_) (Fig. 1a), extracted from the Chinese herb *Curcuma wenyujin*, has been proven to be effective against tumors^18^,^19^,^20^,^21^,^22^ and is approved by China’s State Food and Drug Administration for treating tumors^23^. As a natural plant extract with fewer side effects and strong bone marrow protection, β-elemene is widely applied in the field of cancer chemotherapy^20^,^24^,^25^,^26^. However, the mechanism underlying its anti-tumor effects and how to obtain optimum therapeutic effects of combinational β-elemene and chemotherapy are not well understood.

In our previous research, we found that β-elemene could enhance the anti-HCC effect of oxaliplatin *in vitro* (not published), but how β-elemene enhances this effect remains unclear. The aim of this study was to address the issue of synergism between β-elemene and oxaliplatin in their anti-HCC effect and to elucidate the underlying molecular mechanism for the enhancement of the anti-cancer effect of oxaliplatin. On the basis of our preliminary findings, the effects of β-elemene or oxaliplatin alone and the synergistic effects of the two drugs *in vitro* and *in vivo* were evaluated. We found that β-elemene dramatically reinforced oxaliplatin activity by increasing the amount of intercellular platinum through CTR1 stabilization. Our findings indicate that a new oxaliplatin-based regimen in combination with β-elemene may provide clinical efficacy.

## Results

### Effects of oxaliplatin with or without β-elemene on human HCC cell line proliferation

**Figure 1:**
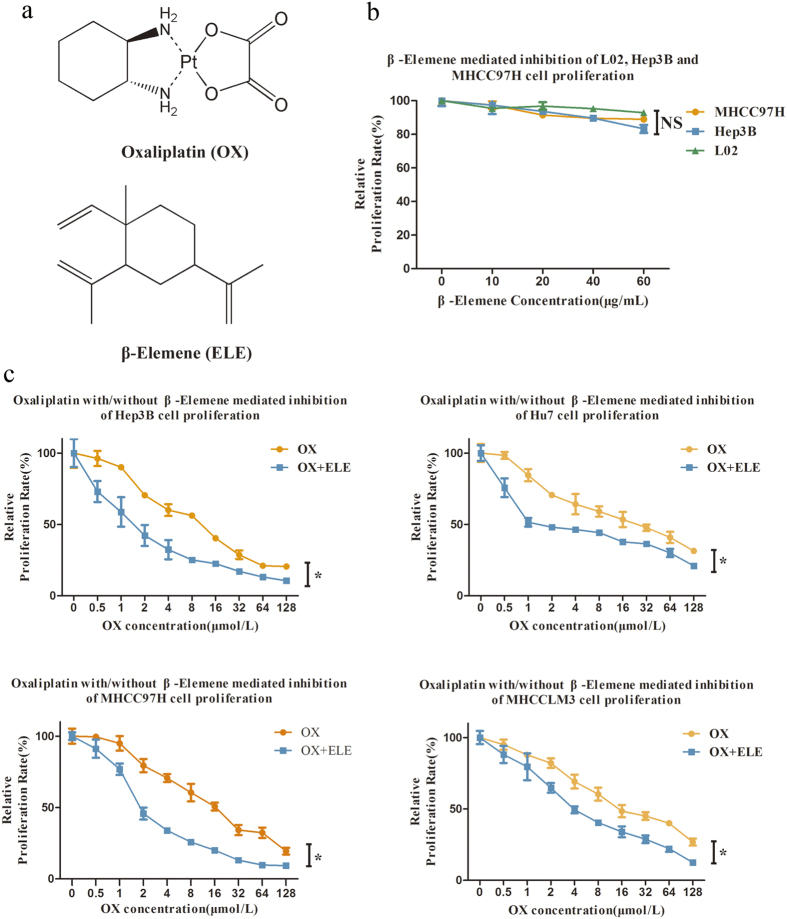
Antitumor activity of oxaliplatin (OX) and/or β-elemene (ELE) applied in human hepatocellular carcinoma cell lines. (**a**) Chemical structures of oxaliplatin and β-elemene. (**b**) Effects of β-elemene on *in vitro* cytotoxicity in normal human liver cell line L02 and HCC cell lines MHCC97H and Hep3B. (**c**) β-elemene increases oxaliplatin cytotoxicity and enhances oxaliplatin sensitivity in human hepatocellular carcinoma cells, as determined by CCK8 assay. (*p < 0.05).

As shown in Fig. 1B, the anti-cancer activity of β-elemene was first measured in normal human liver cell line L02 and HCC cell lines MHCC97H and Hep3B. With increasing β-elemene concentration or incubation time, the percentage of cell viability showed no significant decrease in these cell lines. β-elemene displayed no cytotoxicity toward normal human liver cells. For HCC cells, at concentrations less than 60 μg/mL, β-elemene resulted in no apparent anti-proliferative effects in L02, MHCC97H and Hep3B(Fig. 1b), and similar results were found in two other common HCC cell lines, Huh7 and MHCCLM3 (Supplementary Fig. S1a).

To investigate whether β-elemene could enhance the anti-proliferative effect of oxaliplatin, several HCC cell lines with different malignant potentials were simultaneously treated with oxaliplatin and β-elemene and assessed *in vitro* by CCK8 assay (Fig. 1c). The results showed that oxaliplatin treatment alone (from 0.5 to 128 μm) resulted in a dose-dependent decrease of proliferation in adoptive HCC cell lines (the IC_50_ values for Hep3B, Huh7, MHCC97H, and MHCCLM3 cells at 96 h were 10.00 ± 0.67 μM, 23.24 ± 3.09 μM, 16.41 ± 1.11 μM, and 21.32 ± 1.67 μM, respectively). When combined with β-elemene at 60 μg/mL, the anti-proliferative effects of oxaliplatin increased significantly, showing a striking decrease of IC_50_ to 1.79 ± 0.84 μM, 3.27 ± 0.72 μM, 2.54 ± 0.27 μM, and 5.73 ± 0.74 μM , respectively (Table 1; *p* < 0.01). The values of the dose modifying factor [DMF = IC_50_ (oxaliplatin alone)/IC_50_ (60 μg/mL β-elemene + oxaliplatin)]^27^,^28^ expressing the intensity of enhancement were 5.59, 7.11, 6.46, and 3.72 in Hep3B, Huh7, MHCC97H, and MHCCLM3 cells, respectively. These results suggest that β-elemene and oxaliplatin may act synergistically to enhance cytotoxicity in HCC cell lines.

### Evaluation of synergistic anti-tumor effect between oxaliplatin and β-elemene

To test the combination effect, the HCC cell lines were simultaneously treated with β-elemene and oxaliplatin and the coefficient of drug interaction (CDI) was calculated according to the equation: CDI = AB/(A × B)^29^,^30^,^31^. As expected from the CCK8 assay results, CDI indicated the combination of oxaliplatin and β-elemene yielded synergistic interactions across a wide concentration range (CDI < 1; Table 2). Moreover, for the concentrations applied to the MHCC97H cell line, the CDI value was consistently <0.7, which reveals a significant synergistic effect; the synergistic effect was most prominent (CDI = 0.45) when 60 μg/mL β-elemene was combined with 16 μm oxaliplatin.

### Platinum accumulation and platinum-DNA binding

**Figure 2:**
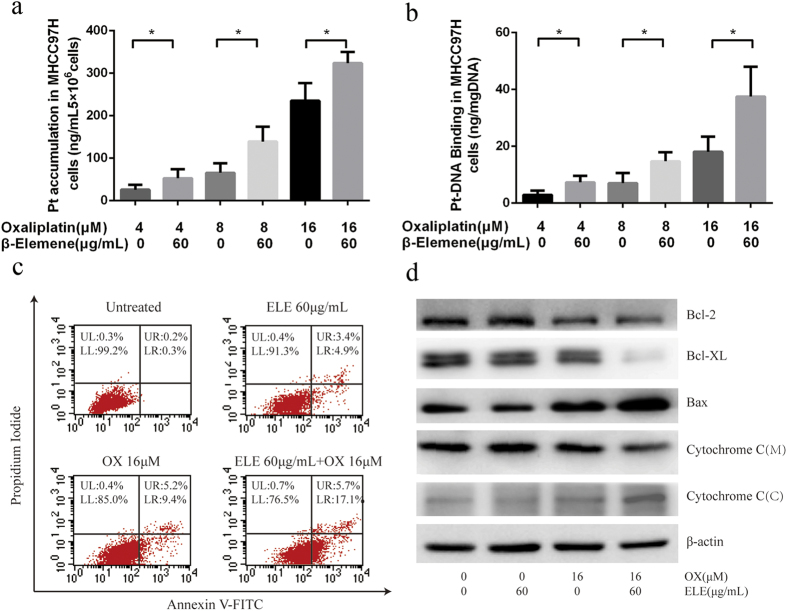
Effect of β-elemene on platinum intake and oxaliplatin-induced apoptosis of MHCC97H cells. (**a**) Cellular platinum accumulation in the MHCC97H cell line with treatment of oxaliplatin alone and oxaliplatin with β-elemene. (*p < 0.05) (**b**) Platinum-DNA binding levels in the MHCC97H cell line due to treatment with oxaliplatin alone and in combination with β-elemene. (*p < 0.05) (**c**) β-elemene promotes Oxaliplatin-induced apoptosis in MHCC-97H cells as assessed by flow cytometric analysis. MHCC97H cells were exposed to oxaliplatin alone or in combination with β-elemene (60 μg/mL) for 48 h. UL upper left, represents necrotic cells (%); UR upper right, represents late apoptotic cells (%); LL lower left, represents viable cells (%); LR lower right, represents early apoptotic cells (%). (**d**) β-elemene increases oxaliplatin-induced changes in protein levels of apoptotic-related molecules in human HCC cells. MHCC97H cells were treated with β-elemene (60 μg/mL), or 16 μm oxaliplatin alone or in combination with β-elemene (60 μg/mL) for 48 hr. Total protein extracts were prepared from cells, separated by SDS-PAGE, and transferred to nitrocellulose membranes. β-actin was used as an internal control. M: mitochondria; C: cytosol.

To determine whether the presence of β-elemene at normal levels resulted in an enhancement in the uptake of oxaliplatin, the level of intracellular platinum in MHCC97H cells was determined after 24-h treatment with different oxaliplatin concentrations with or without β-elemene (Fig. 2a). The total cellular platinum accumulation and DNA-bound platinum was measured by ICP-MS. Exposure to 60 μg/mL β-elemene increased the intracellular accumulation of platinum at all concentrations tested. The 2.25-fold (25.50 ± 6.64 vs 57.50 ± 7.83 ng/mL 5 × 10^6^ cells), 2.13-fold (65.10 ± 12.85 vs 138.70 ± 20.20 ng/mL 5 × 10^6^ cells), and 1.38-fold (234.80 ± 23.90 vs 323.50 ± 14.95 ng/mL 5 × 10^6^ cells) increases observed with 4, 8, and 16 μm oxaliplatin, respectively, were statistically significant. Similar results were found in Huh7 and Hep3B (data not shown).

Because the action of platinum drugs is associated with their binding with DNA and exerting cytotoxic effect mostly through DNA damage, the DNA-bound platinum levels in MHCC97H were also measured. β-elemene significantly increased oxaliplatin-generated platinum-DNA adduct in MHCC97H cells. As compared to oxaliplatin alone, the combination of β-elemene with oxaliplatin generated approximately 2.50-, 2.10-, and 2.08-fold more DNA adducts in MHCC97H cells with 4, 8, and 16 μm oxaliplatin, respectively (Fig. 2b). Similar results were found in Huh7 and Hep3B (data not shown).

### β-elemene promotes oxaliplatin-induced apoptosis in MHCC97H cells

Platinum-based agents including oxaliplatin exert their anti-tumor effect by stimulating apoptosis. To investigate the effect of β-elemene on oxaliplatin-induced apoptosis, two-color flow cytometry of MHCC97H cells stained with Annexin V-FITC and propidium iodide (PI) was performed. MHCC97H cells staining with Annexin V-FITC resolved early apoptotic cells, whereas MHCC97H cells staining with Annexin V-FITC and PI resolved late apoptotic and necrotic cells. The percentages of early apoptotic cells after treatment with oxaliplatin plus 60 μg/mL β-elemene for 48 hr was about 2-fold the number after treatment with oxaliplatin alone (Fig. 2c).

Apoptosis triggered by oxaliplatin plus β-elemene was further confirmed by Western blot analysis in MHCC97H cells. The protein levels of apoptotic-related molecules in human HCC cells cells after exposure to oxaliplatin alone or oxaliplatin plus β-elemene for 48 hr were analyzed by Western blotting. Western blot analysis revealed that the oxaliplatin/β-elemene combination greatly reduced the expression of anti-apoptotic protein Bcl-2 and Bcl-XL and upregulated the expression of pro-apoptotic protein BAX. The oxaliplatin/β-elemene combination, but not β-elemene treatment alone, promoted cytochrome c release from the mitochondria into the cytosol (Fig. 2d).

### β-elemene enhances anti-HCC effect of oxaliplatin by preventing oxaliplatin-induced degradation of hCTR1

**Figure 3:**
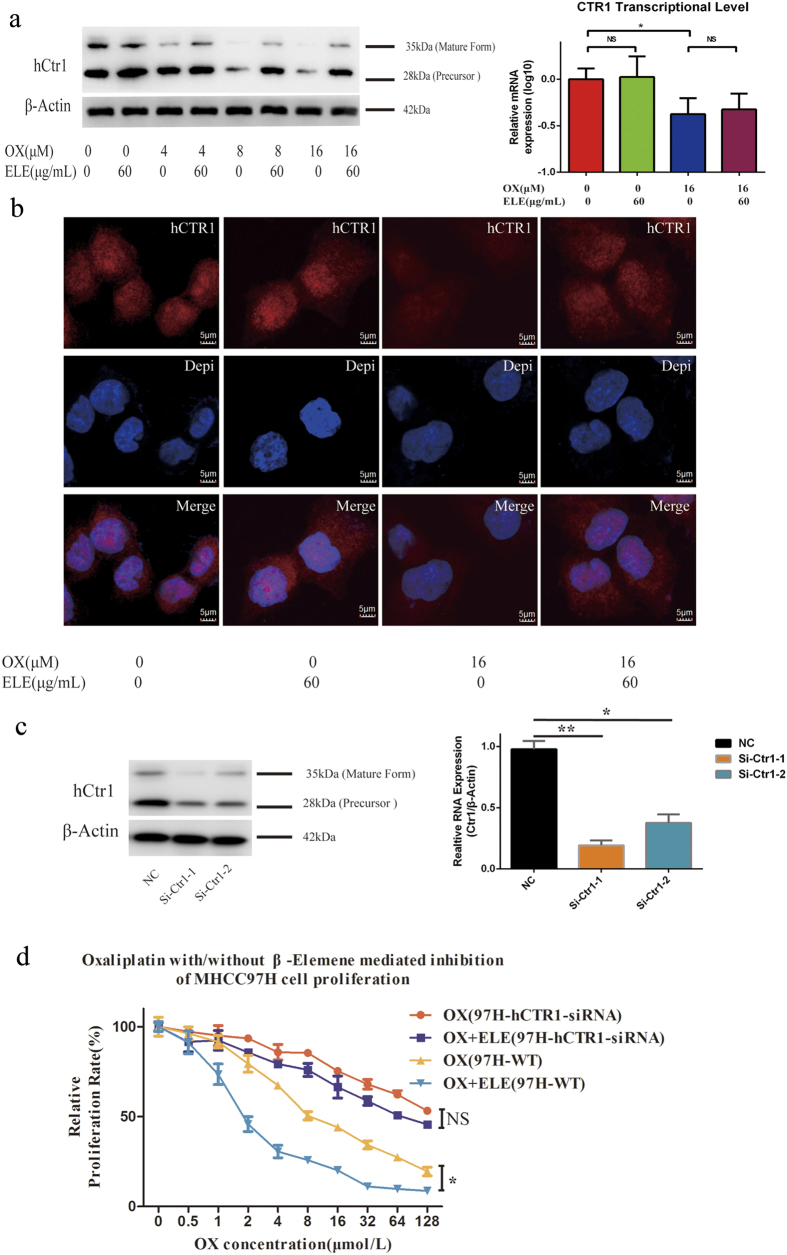
Effect of β-elemene (ELE) on the level of hCTR1 in the presence of oxaliplatin (OX). (**a**) Western blot analysis of hCTR1 expression. MHCC97H cells exposed to various oxaliplatin concentrations with or without 60 μg/mL β-elemene for 12-h exposure. Graph shows ratio between hCTR1 and β-actin expression. CTR1 transcriptional levels was measured by real-time PCR after MHCC97H cells were treated with and without β-Elemene for 48 h. Data shown are mean (± S.D.) from three independent experiments.*p < 0.05, compared with control cells. (**b**) Confocal digital microscopic analyses of hCTR1 levels in MHCC97H cells exposed to oxaliplatin (0, 16 μm) with or without 60 μg/mL β-elemene for 2 h. Red, hCTR1; blue, nucleus stained with 4,6-diamidino-2-phenylindole. Results are representative of three independent experiments; image intensity was normalized to cells stained only with secondary antibody. (**c**) MHCC97H cells were transfected with hCTR1-siRNA for 24 h. Cell lysates were then harvested and subjected to Western blot and RT-PCR analysis. (**d**) Transfected MHCC97H cells were more resistant to oxaliplatin and displayed no cytotoxicity difference between oxaliplatin plus β-elemene and oxaliplatin alone. (*p < 0.05, ** p < 0.01).

The mechanism that allows β-elemene to enhance oxaliplatin’s anti-HCC effect was further studied by focusing on the oxaliplatin processing pathway. Western blot analysis was performed to measure the expression levels of copper transporters in MHCC97H cells. The expression level of the copper influx transporter CTR1 decreased with the increasing concentration of oxaliplatin^32^. In addition, an increase in CTR1 expression was found for oxaliplatin plus β-elemene relative to oxaliplatin alone. We applied real-time reverse-transcription PCR to perform comparative analysis of CTR1 differential expressions of different treatment groups. The data showed that oxaliplatin-induced down-regulation of CTR1 was independent of transcriptional and translational events (Fig. 3a). Confocal microscopic images suggested that CTR1 decreased sharply upon exposure to oxaliplatin and that this trend could be reversed by simultaneous treatment with β-elemene (Fig. 3b).

To further confirm the key role of CTR1 in the synergistic effect of β-elemene with oxaliplatin, we generated two CTR1-specific siRNAs to silence the endogenous CTR1 (si-hCTR1) of HCC cells to observe the different response of HCC cells to the regimen. Si-CTR1#1, inducing a >80% decrease in CTR1, was adopted for further study (Fig. 3c). As shown in Fig. 3d, transfected MHCC97H cells were more resistant to oxaliplatin and displayed no cytotoxicity difference between oxaliplatin combined with β-elemene and oxaliplatin alone (*P* = 0.8135), indicating the important role of CTR1stabilization induced by β-elemene on the anti-HCC effect of oxaliplatin. A similar result was found in the HCC cell line Hep3B (Supplementary Fig. S1).Thus, as documented by three different analytic techniques, β-elemene was able to prevent oxaliplatin-induced degradation of CTR1 in a manner similar to some proteasome inhibitors like bortezomib^33^.

### β-elemene enhanced the anti-tumor activity of oxaliplatin in a HCC xenograft model

**Figure 4:**
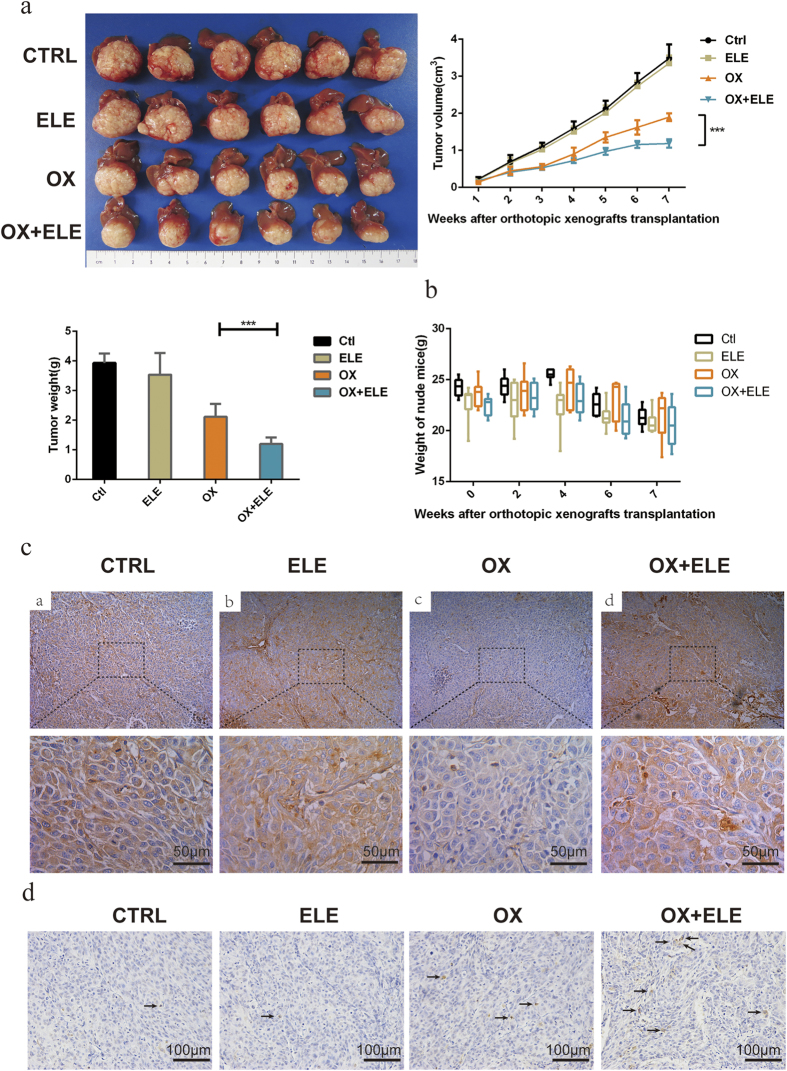
The effects of oxaliplatin (OX) with or without β-elemene (ELE) on *in vivo* tumor growth in an orthotopic implantation mouse model of MHCC97H cells and on CTR1 protein expression in tumor tissues. Oxaliplatin with β-elemene suppressed tumor growth in orthotopic implantation mouse models of MHCC97H cells. (**a**) Tumor volumes and weight in the orthotopic implantation model mice at week 7. (**b**) The weight in the orthotopic implantation model mice at the indicated time points. (***p < 0.001) (**c**) Representative examples of immunohistochemistry staining for CTR1 protein from MHCC97H xenografts (400 × magnification; scale bars, 50 μm). (**d**) Representative examples of tumor xenografts stained with apoptosis by TUNEL assay (positive cell labelled with arrows; 400 × magnification; scale bars, 50 μm).

To confirm these findings, we further examined the influence of β-elemene on the anti-HCC effect of oxaliplatin on *in vivo* HCC growth. Compared with tumor growth in the PBS treatment group, β-elemene alone made no difference, but the enhancement of anti-tumor activity was observed when mice were treated with oxaliplatin plus β-elemene relative to oxaliplatin alone (Fig. 4a, Supplementary Fig. S2). Concurrently, β-elemene alone showed no significant influence on weight and morbidity of nude mice (Fig. 4b). Immunohistochemical analysis of HCC samples further verified the molecular mechanism underlying β-elemene’s ability to prevent oxaliplatin-induced degradation of hCTR1: the expression of hCTR1 in the oxaliplatin treatment group decreased significantly, but in the combined β-elemene treatment group the expression of hCTR1 reversed to the level of PBS treatment (Fig. 4c). To further validate β-elemene promoting oxaliplatin-induced apoptosis *in vivo*, we examined the HCC samples by TUNEL (Fig. 4d). The distribution of apoptotic cells was evaluated by counting positively labeled cells in randomly selected five microscopic fields. The apoptotic index was presented as the percentage of positive cells for each zone. The untreated group (0.60 ± 0.24) and the β-elemene treatment group(0.80 ± 0.37) showed lower apoptotic indices, but the oxaliplatin treatment group(2.6 ± 0.51) and the combined β-elemene treatment group(6.0 ± 0.32) showed higher apoptotic indices, as well as statistically significant difference between the latter two groups(p < 0.001). Taken together, these results indicate that β-elemene, via the major platinum influx transporter, potently enhanced oxaliplatin’s anti-HCC effect *in vitro* and *in vivo* by preventing oxaliplatin-induced degradation of hCTR1.

## Discussion

Due to the poor liver function and chemotherapy tolerance, no successful chemotherapy regimen to prolong the survival of HCC patients has been widely accepted^34^,^35^. The efficacy of oxaliplatin in treating otherwise fatal diseases has been established based on 30 years of experience^36^. Recently, this agent has gained increasing interest when used in combination with resistance modulators or new molecularly targeted drugs for the purposes of lowering doses of chemotherapeutic agents, reducing side effects, and increasing treatment efficacy^15^. Our institute has explored the role of autophagy as a resistance mechanism of oxaliplatin treatment and the potential therapeutic value of combining oxaliplatin and autophagy inhibitors to treat HCC^37^. In the present study, we assessed the combination effect of oxaliplatin and the botanical extract β-elemene on human HCC cell lines. The synergism of oxaliplatin and β-elemene was determined *in vivo* and *in vitro*, suggesting greater cellular accumulation of oxaliplatin and a possible interaction between β-elemene and the influx transporter of oxaliplatin. The results also indicated the pharmacologic advantage of β-elemene prompting more oxaliplatin accumulation than when oxaliplatin is used alone. These insights into the interaction between oxaliplatin and β-elemene will help in the development of a novel strategy to improve the efficacy of HCC chemotherapy and overcome acquired oxaliplatin resistance.

Many preclinical studies have suggested that HCC is chemotherapy-resistant, with an objective response rate to systemic chemotherapy that is normally less than 10%^38^,^39^. Hence, it is imperative to identify and validate novel therapeutic strategies for improving chemo-resistance in HCC research. Recently, the promising anti-cancer activity of many plant extracts has aroused researchers’ interests. β-elemene has been shown to have broad-spectrum anti-tumor effects on many types of cancer and low toxicity in patients^19^,^20^,^21^,^40^,^41^,^42^,^43^, although the precise mechanism of β-elemene remains to be elucidated. Previous studies showed β-elemene could induce G2/M phase arrest and promote apoptosis in ovarian cancer cells and non-small cell lung cancer cells^20^,^21^. Another study demonstrated that β-elemene could inhibit breast cancer cell growth via an inhibitory effect on mTOR and by inducing autophagy^44^. In lung carcinoma β-elemene may induce the alteration of cell membrane permeability, which has the potential to result in enhanced cellular uptake of taxanes^26^. In ovarian cancer β-elemene may reduce DNA repair activity and enhance cisplatin cytotoxicity by blocking the activation of PI3K/JNK signaling pathway^43^, and in prostate cancer β-elemene has been shown to enhance the cisplatin-induced apoptosis via mitochondrial activation of the caspase-mediated apoptotic pathway^24^.

**Figure 5:**
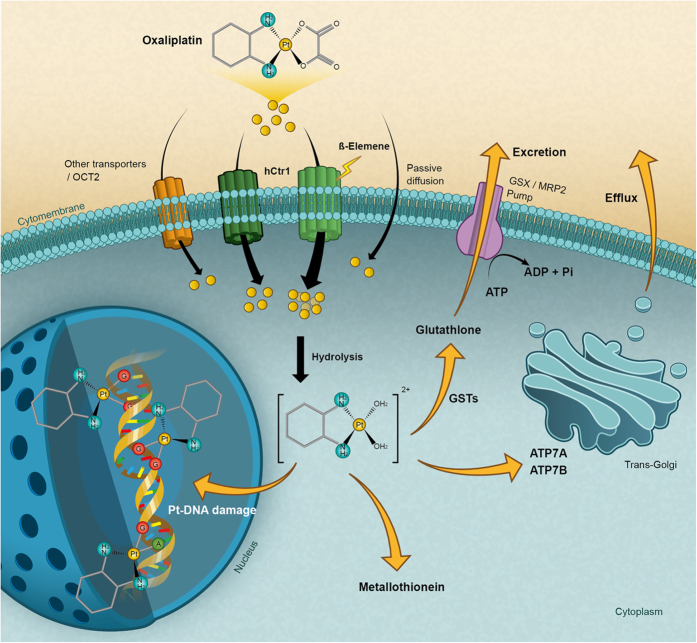
A hypothetical schematic of the contribution of β-elemene to oxaliplatin inhibition of HCC via activation of the CTR1. Platinum drugs enter cells either by transporters, such as CTR1, or by passive diffusion. β-elemene increases the intercellular platinum amount by stabilizing CTR1, counteracting the degradation of CTR1 upon oxaliplatin treatment. Once inside cells, oxaliplatin is hydrolyzed by the addition of water molecules to form a chemically reactive aqua species, which reacts with species containing high sulfur levels from many cysteine or methionine amino acids including tripeptide glutathione or metallothioneins. Oxaliplatin can be exported from cells through the copper exporters ATP7A and ATP7B or through the glutathione S-conjugate export GS-X pump. Once the activated aqua oxaliplatin species has entered the nucleus, prior binding to the nitrogen at position 7 of guanine occurs and causes DNA damage.

We investigated the anti-tumor effect of β-elemene in human HCC *in vivo* and *in vitro*, but found little effect of β-elemene on HCC. However, we showed for the first time that β-elemene could dramatically enhance oxaliplatin-induced cytotoxicity in HCC cells. Furthermore, we explored the mechanisms of β-elemene increasing oxaliplatin sensitivity in human HCC and found that β-elemene promoted oxaliplatin importation by upregulating expression of the copper influx transporter CTR1, which was degraded significantly upon oxaliplatin treatment^45^,^46^. This is the first demonstration that β-elemene could act on CTR1 to deliver a sufficient amount of platinum to reach the target DNA (Fig. 5).

CTR1 was originally recognized as a multispanning plasma membrane protein that was closely related to copper transport into yeast cells^47^ and was proven to be elementary for stimulating copper accumulation in mammalian cells^48^,^49^,^50^. Subsequent research found that CTR1 markedly influenced the cellular accumulation of platinum-containing drugs, which strengthened the value of CTR1 in platinum-based chemotherapy^51^,^52^. Emerging evidence indicated that the degradation of the CTR1 protein localized in plasma membrane and cytoplasm was triggered by platinum drugs^45^,^46^. Consistent with previous research, in this study the loss of CTR1 protein upon oxaliplatin treatment was observed both by Western blot and immunofluorescence confocal microscopy analysis in MHCC97H cells. β-elemene could counteract the loss of CTR1 expression and enhance the activity of platinum drugs, as demonstrated by the fact that β-elemene had no effect on CTR1 expression and platinum influx when MHCC97H cells were transfected by CTR1 siRNA, indicating the key role of CTR1 in the synergistic effects of β-elemene plus oxaliplatin. Unexpectedly, the ability of β-elemene to regulate CTR1 expression depends on oxaliplatin, as β-elemene alone had little effect on the expression of CTR1. CTR1 shows to control the tumor cell accumulation and cytotoxic effect of cisplatin, carboplatin, and oxaliplatin and cisplatin can trigger the down-regulation of CTR1 via a process that involves ubiquitination and proteosomal degradation^14^. Our data shows that oxaliplatin-induced down-regulation of CTR1 are independent of transcriptional and translational events. So we hereby hypothesize that oxaliplatin can influence the processes of ubiquitination of CTR1. In addition, structure-function analysis of CTR1 indicated that N-terminal Met-rich motifs were critical for copper and platinum transport binding to the N terminus^53^,^54^,^55^. Whether β-elemene regulates CTR1-mediated oxaliplatin transport by targeting the N terminus remains to be clarified and deserves further study.

We found this powerful combination is therapeutically effective, providing a novel, safe and practical form of chemotherapy. However, the mechanism of β-elemene stabilizing CTR1 need further study. We plan to study on weather β-elemene act on the ubiquitination of CTR1 in the next step. We also design a dose response study *in vivo* including the different doses of β-elemene to testify if there is the dose response relationship between β-elemene and the efficacy.

In summary, we found that β-elemene dramatically reinforced the anti-proliferative effect of oxaliplatin on HCC both *in vitro* and *in vivo*. This synergism results from platinum accumulation and increasing of platinum-DNA adduct. Moreover, the CTR1 siRNA-based analysis showed that β-elemene increases the intercellular platinum amount by stabilizing CTR1, counteracting the degradation of CTR1 upon oxaliplatin treatment. These data support the concept that an oxaliplatin-based regimen in combination with β-elemene can result in a more powerful anti-HCC effect, and it deserves further evaluation in future clinical settings.

## Methods

### Chemicals and reagents

β-elemene (99.2% purity) was purchased from the Chinese National Institutes for Food and Drug Control, with a molecular formula of C_15_H_24_ and molecular weight of 204.35. Injectable solution of β-elemene (#081152) was purchased from Dalian Holley Kingkong Pharmaceutical Co., Ltd (Liaoning, China). Oxaliplatin was purchased from Sigma Chemical Company (#O9512, St. Louis, MO, USA). For Western blotting and/or immunochemistry, anti-CTR1/SLC31A1 antibody was purchased from Abcam (#ab129067, San Francisco, CA, USA), anti-β-actin was purchased from Santa Cruz Biotechnology (#sc-47778, Paso Robles, CA, USA), and goat-anti-rabbit IgG (#cw0103A) and goat-anti-mouse IgG (#cw0102) conjugated to horseradish peroxidase were purchased from Cwbiotech (Beijing, China). Primary antibodies of apoptotic-related molecules were purchased from Proteintech (Bcl-2(#12789-1-AP, Chicago, IL, USA), Bcl-XL (#12783-1-AP, Chicago, IL, USA), BAX (#50599-2-AP, Chicago, IL, USA), Cytochrome C(#10993-1-AP, Chicago, IL, USA)). Cell proliferation was measured using the Cell Counting Kit-8 assay (#ck04, Dojindo, Kumamoto, Japan).

### HCC cell lines

Two HCC cell lines (MHCC97H and HCCLM3) that were established from the same parent human HCC cell line were obtained from the Liver Cancer Institute, Fudan University, Shanghai, China. The HCC cell lines Hep3B and Huh7 were purchased from Shanghai Institute of Cell Biology, Shanghai, China. All cell lines were maintained and cultured in high-glucose Dulbecco’s modified Eagle’s medium (DMEM) (Gibco BRL, Grand Island, NY, USA) supplemented with 10% fetal bovine serum (Gibco BRL), which were incubated at 37 °C in a humidified incubator containing 5% CO_2_.

### Cell transfections

MHCC97H cells were seeded at a density of 5 × 10^5^ cells/well in 6-well polystyrene microplates and incubated until they reached 50% confluence. Small interfering RNA (siRNA) was purchased from Biomics (Nantong, China), including hs-CTR1-si-1,2 and a negative control (si-NC) (Supplementary Table S1). Cells were transiently transfected with siRNA for 6 h at a final concentration of 50 nM with Lipofectamine 2000 transfection reagent (Life Technologies-Invitrogen, Shanghai, China) according to the manufacturer’s instructions. After the transfection in serum-free DMEM for 6 h, the cells were cultured with DMEM containing 10% fetal bovine serum for 48 h, followed by quantification of mRNA using real-time qPCR analysis and Western blot analysis.

### CCK8 assay and combination studies

A CCK8 assay was used to detect the absorbance at a wavelength of 450 nm as the OD value. Inhibition concentration 50 (IC_50_) values were calculated and converted into dose modifying factors [DMF = IC_50_ (oxaliplatin alone)/IC_50_ (60 μg/mL β-elemene + oxaliplatin)]. The nature of drug interaction was analyzed by the coefficient of drug interaction (CDI)^29^,^30^,^31^, which was calculated as CDI = AB / (A × B). According to the absorbance of each group, AB is the ratio of the two-drug combination group to the control group, and A or B is the ratio of the single agent group to the control group. Thus, a CDI value of <1, = 1, or >1 indicates that the drugs are synergistic, additive, or antagonistic, respectively.

### Platinum cellular accumulation and platinum-DNA binding studies

MHCC97H cells were seeded in 100-mm culture dishes at a density of 5 × 10^6^ cells per well in aliquots of 10 mL complete culture medium (see *HCC Cell lines*). Cells in the exponential growth phase were exposed to different concentrations (for details see *Results*) of oxaliplatin with or without β-elemene at 37 °C in a humidified incubator containing 5% CO_2_ for 24 h. After incubation, cells were washed with ice-cold PBS, harvested, and pelleted by centrifugation at 400 × *g* and at 4 °C for 15 min. The cell pellets were dissolved in 70% nitric acid at 65 °C for at least 2.5 h. Distilled water containing 10 ppb of iridium (Sigma) and 0.1% Triton X-100 was added to the samples to dilute the nitric acid to 7%. The platinum content was measured by inductively coupled plasma mass spectrometry (ICP-MS) in the State Key Laboratory of Estuarine and Coastal Research at the East China Normal University at Shanghai.

Genomic DNA was isolated from the cell pellets using a Wizard Genomic DNA Purification kit (Promega, Madison, WI, USA) following the manufacturer’s instructions. The platinum-binding genomic DNA was analyzed by ICP-MS.

### Flow cytometric analysis of apoptosis

To quantify apoptosis, MHCC97H cells were treated with oxaliplatin (16 μm), either alone or in combination with β-elemene (60 μg ⁄ml), for 48 hr. After treatment, the cells were harvested by trypsinization, washed twice with PBS and stained with Annexin V: FITC Apoptosis Detection Kit (#556547, BD Bioscience, Eugene, NJ, USA), following the manufacturer’s protocol. Flow cytometric analysis of the stained cells was performed by BD FACSCalibur with CellQuest software (BD Bioscience, San Jose, CA).

### Animal treatment

BALB/c nu/nu mice (aged 4 weeks and weighing approximately 20 g) were purchased from the Shanghai Institute of Materia Medica and maintained under standard pathogen-free conditions in the experimental animal center of Zhongshan Hospital, Fudan University. The animal care and experimental protocols were in accordance with the guidelines established by the Shanghai Medical Experimental Animal Care Commission. Ethical approval was obtained from the Zhongshan Hospital Research Ethics Committee.

Orthotopic implantation of the human HCC cell line MHCC97H in the BALB/c mice was carried out as described previously^56^. Seven days after orthotopic implantation, 24 BALB/c nu/nu mice bearing orthotopic xenografts were randomly divided into a control group, which was treated by intraperitoneal injection with 0.1 mL 5% glucose solution twice a week; β-elemene group, which was treated by intraperitoneal injection with 0.1 mL β-elemene (45 mg/kg) twice a week; oxaliplatin group, which received intraperitoneal injection with 0.1 mL oxaliplatin (5 mg/kg) twice a week; and oxaliplatin plus β-elemene group, which was treated by intraperitoneal injection with 0.1 mL oxaliplatin (5 mg/kg) plus 0.1 mL β-elemene (45 mg/kg) twice a week. Tumor growth and mice weight were evaluated twice a week, and mice were sacrificed after 7 weeks.

Tumors, livers, and lungs were removed, fixed in 4% formalin fixative overnight at 4 °C, and embedded in paraffin. Consecutive 5-μm thick sections of tumor and liver were made for immunohistochemistry. The expression of CTR1 in tumors was measured in serial tissue sections of each group by immunohistochemistry.

### Immunohistochemistry

Immunohistochemistry staining of paraffin sections was performed using a two-step protocol, using Novolink Polymer Detection System (NovoLink. TM. Detection System, Leica, UK) and the GTVision II Detection Kit with peroxidase/DAB, rabbit/mouse (#gk500506A, Gene Tech, Shanghai, China). After antigen retrieval, the slices were incubated with the primary antibody (#sc-66847, CTR1, Santa Cruz, CA, USA) overnight at 4 °C, followed by incubation with the secondary antibody (#gk500506A, Gene Tech) at 37 °C for 30 min. The sections were stained with DAB and counterstained with hematoxylin (Dako, Glostrup, Denmark), dehydrated in ethanol, mounted in dimethyl benzene, and placed under a coverslip. Apoptotic cells were visualized with an enzymatic reaction using the TUNEL method. TUNEL staining with an *in situ* cell death detection kit (#11684817910, Roche Molecular Biochemicals, Indianapolis, IN, USA), following the manufacturer’s protocol.

### Western blot analysis and immunofluorescence

Total protein was extracted by cell lysis buffer supplemented with protease inhibitor. Protein samples were separated using 10% sodium dodecyl sulfate-polyacrylamide gel electrophoresis and electrotransferred onto polyvinylidene fluoride membranes (Millipore, Billerica, MA, USA). After blocking with 5% non-fat milk in TBS-T, membranes were incubated with the primary antibody (#ab129067, CTR1, Abcam, San Francisco, CA, USA). Protein was detected with Image Acquisition using ImageQuant LAS 4000 (Pittsburg, PA, USA).

For immunofluorescence assays, cells were cultured on glass slides with cell abundance of 30% in 6-well polystyrene microplates. The cells on glass slides were fixed with 4% formaldehyde and then permeabilized with PBS containing 0.2% Triton X-100 after washing three times in PBST. After blocking with 1% BSA in PBS, the cells were incubated with primary antibody (#sc-66847, CTR1, Santa Cruz, CA, USA) overnight at 4 °C. Cells were washed three times in PBS, followed by incubation with 2 μg/mL Alexa Fluor 594 phalloidin (Invitrogen, Carlsbad, CA, USA) for 1 h at room temperature. Cells were washed with PBS three times, nuclear stained with 5 μg/mL DAPI, and analyzed with a confocal laser scanning microscope (Olympus FluoView FV1000 confocal microscope, Melville, NY, USA).

### Statistical analyses

Statistical analyses were performed with SPSS 19.0 for Windows (SPSS Inc., Chicago, IL, USA). Values of continuous variables are expressed as the mean ± SD. The difference between groups was analyzed using a Student’s *t-*test when comparing only two groups or one-way analysis of variance when comparing more than two groups. Quantitative variable comparisons were analyzed using *χ*^2^ test or Fisher’s exact test. Two-tailed *P* values < 0.05 were considered statistically significant.

## Additional Information

**How to cite this article**: Li, X. *et al.* β-elemene sensitizes hepatocellular carcinoma cells to oxaliplatin by preventing oxaliplatin-induced degradation of copper transporter 1. *Sci. Rep.* **6**, 21010; doi: 10.1038/srep21010 (2016).

## Supplementary Material

Supplementary Information

## Figures and Tables

**Table 1:** β-elemene (60 μg/mL) increases oxaliplatin cytotoxicity and enhances oxaliplatin sensitivity in human hepatocellular carcinoma cells, as determined by CCK8 assay.

Cell lines	Drug	IC_50_(96 h)	*P*-value	Dose-modifying factor
Hep3B	oxaliplatin (μM)	10.00 ± 0.67	0.0016	5.59
	β-elemene+oxaliplatin (μM)	1.79 ± 0.84		
Huh7	oxaliplatin (μM)	23.24 ± 3.09	0.0033	7.11
	β-elemene+oxaliplatin (μM)	3.27 ± 0.72		
MHCC97H	oxaliplatin (μM)	16.41 ± 1.11	0.0003	6.46
	β-elemene+oxaliplatin (μM)	2.54 ± 0.27		
MHCCLM3	oxaliplatin (μM)	21.32 ± 1.67	0.0010	3.72
	β-elemene+oxaliplatin (μM)	5.73 ± 0.74		

IC_50_ value is the concentration of drug required to inhibit cell growth by 50% relative to dilute controls; presented as the mean of eight simultaneous replicates repeated three times. Data are mean ± SD of 3 biological replicates.

**Table 2:** Synergistic effects of oxaliplatin and β-elemene in human hepatocellular carcinoma cells.

	Cell lines	β-elemene (60 μg/mL)			
		Oxaliplatin(2 μm)	Oxaliplatin(4 μm)	Oxaliplatin(8 μm)	Oxaliplatin(16 μm)
CDI	Hep3B	0.78	0.83	0.86	0.81
	Huh7	0.83	0.83	0.75	0.81
	MHCC97H	0.65	0.54	0.48	0.45
	MHCCLM3	0.89	0.80	0.75	0.79

Coefficient of drug interaction (CDI) was calculated with the equation CDI = AB/(A × B) (AB, relative cell viability of the combination; A or B, relative cell viability of the single agent groups). CDI < 1 indicates a synergistic effect; CDI = 1 indicates an additive effect; CDI > 1 indicates an antagonistic effect.
