# Supplementary material for: β-elemene sensitizes hepatocellular carcinoma cells to oxaliplatin by preventing oxaliplatin-induced degradation of copper transporter 1
Source: Sci Rep. 2016 Feb 12;6:21010. doi: 10.1038/srep21010 (PMC4751482; doi:10.1038/srep21010)
Supplement: Supplementary Information [file srep21010-s1.pdf]

β-elemene sensitizes hepatocellular carcinoma cells to oxaliplatin by preventing oxaliplatin-induced degradation of copper transporter 1

Xiaoqiang Li[1,2*], Zhenhai Lin[3*], Bo Zhang[1,2*], Lei Guo[1,2], Shuang Liu[1,2], Hui Li[1,2], Jubo Zhang[1,2], **&** Qinghai Ye[1,2]

Correspondence and requests for materials should be addressed to Q.Y. (email: ye.qinghai@zs-hospital.sh.cn) or J.Z. (email: zhang.jubo@zs-hospital.sh.cn)

*These authors contributed equally to this work.

Supplementary Table S1. Sequences of the forward and reverse primers for si-RNA used in this study

Primer name		Sequence (5'-3')
hs-CTR1-si-1	F	CUCCAACAGUACCAUGCAAdTdT
	R	UUGCAUGGUACUGUUGGAGdTdT
hs-CTR1-si-2	F	CCGGUACAGGAUACUUCCUdTdT
	R	AGGAAGUAUCCUGUACCGGdTdT

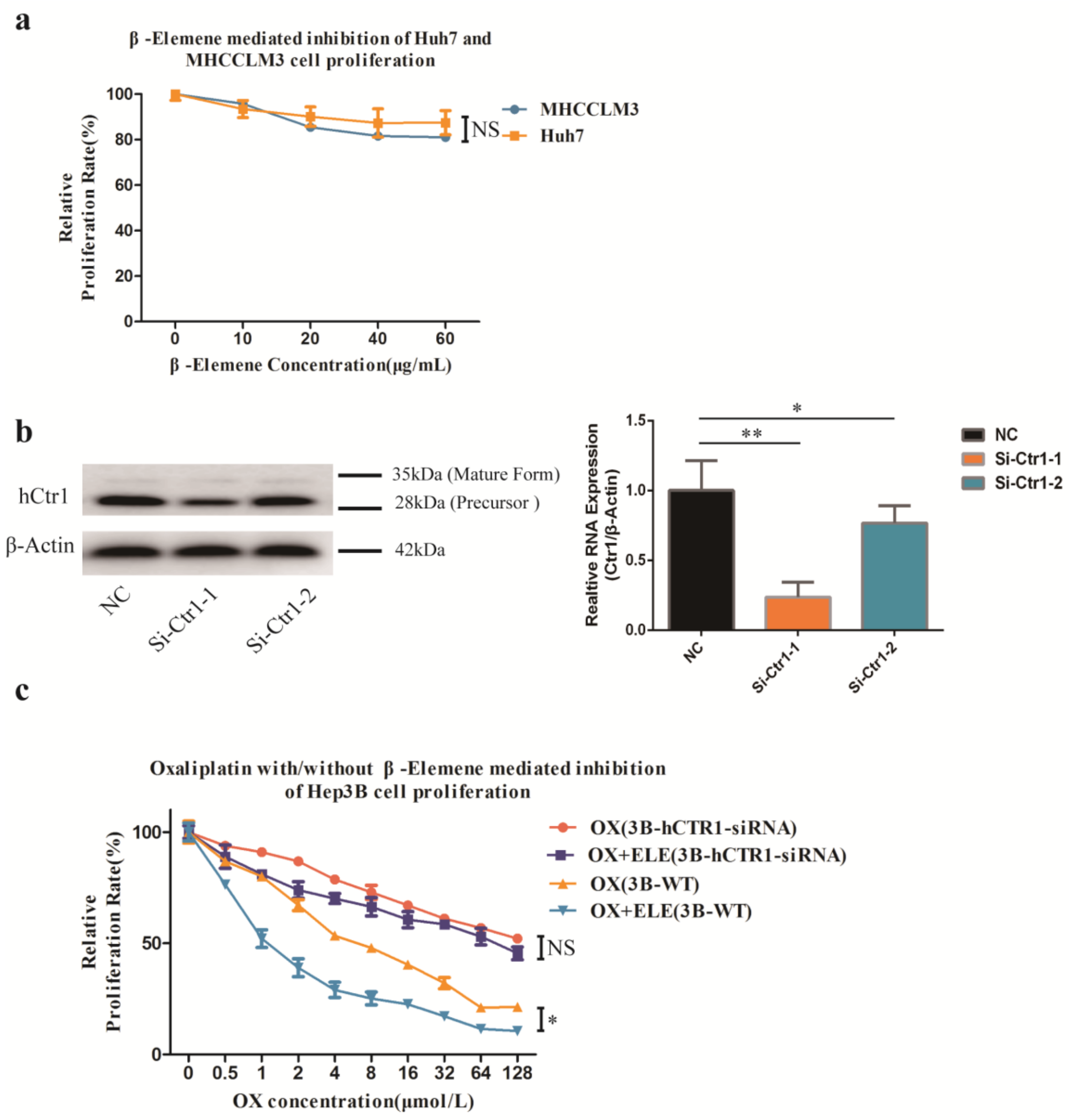

Supplementary Figure S1. Effect of β-elemene (ELE) on the level of hCTR1 in the presence of oxaliplatin (OX). (a) Effects of β-elemene on *in vitro* cytotoxicity in HCC cell lines Huh7 and MHCCLM3. (b) Hep3B cells were transfected with hCTR1-siRNA for 24 h. Cell lysates were then harvested and subjected to Western blot and RT-PCR analysis. (c) Transfected Hep3B cells were more resistant to oxaliplatin and displayed no cytotoxicity difference between oxaliplatin plus β-elemene and oxaliplatin alone. (*$p < 0.05$, **$p < 0.01$).

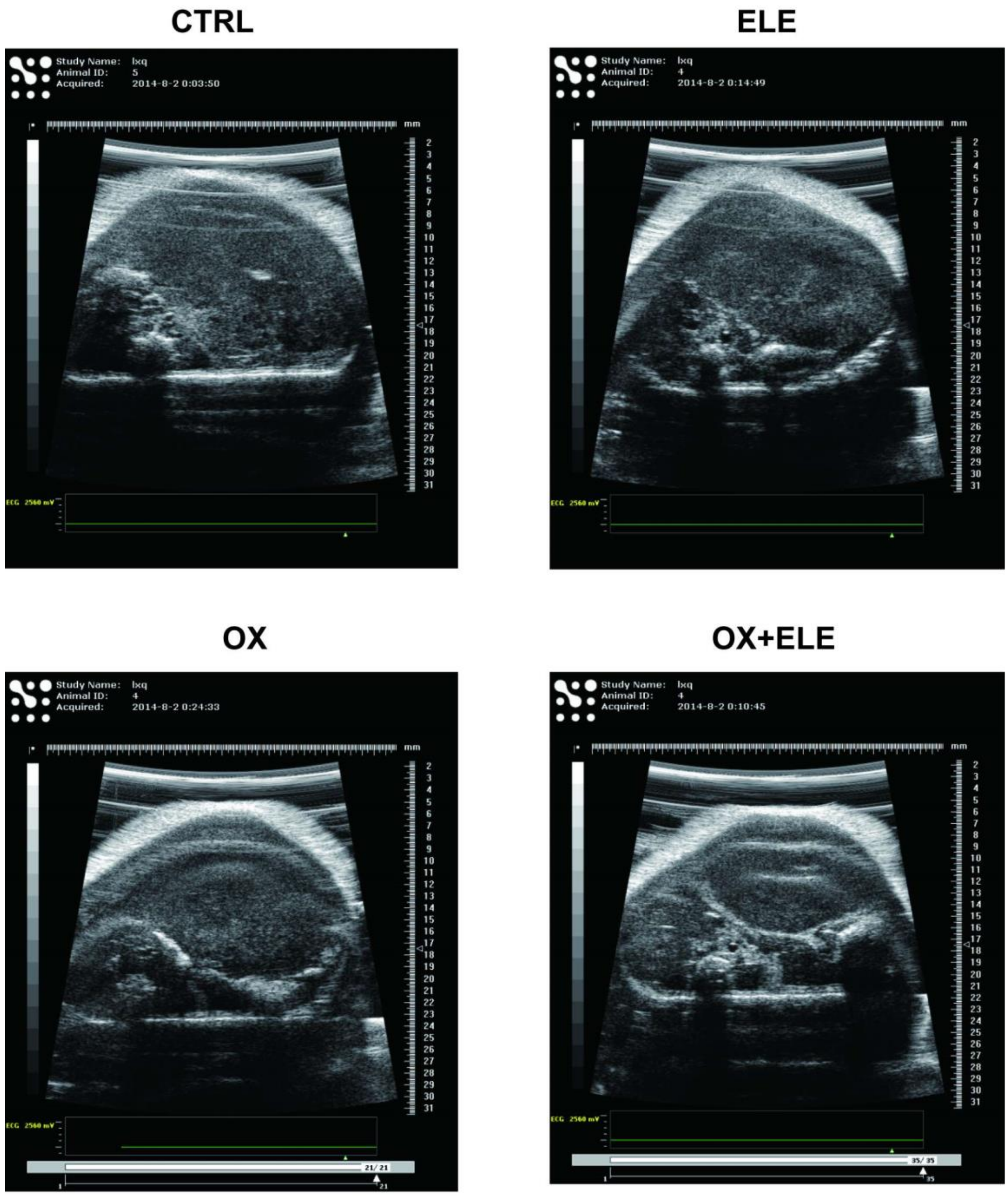

Supplementary Figure S2. Ultrasonography of the orthotopic implantation models at week 6.
